# PR-1-Like Protein as a Potential Target for the Identification of *Fusarium oxysporum*: An In Silico Approach

**DOI:** 10.3390/biotech10020008

**Published:** 2021-04-14

**Authors:** Olalekan Olanrewaju Bakare, Arun Gokul, Marshall Keyster

**Affiliations:** 1 Environmental Biotechnology Laboratory (EBL), Department of Biotechnology, University of the Western Cape, Cape Town 7535, South Africa; Agokul5@gmail.com (A.G.); mkeyster@uwc.ac.za (M.K.); 2 Bioinformatics Research Group, Department of Biotechnology, University of the Western Cape, Cape Town 7535, South Africa

**Keywords:** antimicrobial peptides, pesticides, resistance, algorithms, fungal, proteins, receptors

## Abstract

*Fusarium oxysporum* remains one of the leading causes of economic losses and poor crop yields; its detection is strained due to its presentation in various morphological and physiological forms. This research work sought to identify novel biomarkers for the detection of *Fusarium oxysporum* using in silico approaches. Experimentally validated anti-*Fusarium oxysporum* antimicrobial peptides (AMPs) were used to construct a profile against *Fusarium oxysporum*. The performance and physicochemical parameters of these peptides were predicted. The gene for the *Fusarium oxysporum* receptor protein PR-1-like Protein, Fpr1, was identified and translated. The resulting protein model from the translation was then validated. The anti-*Fusarium oxysporum* AMPs and *Fusarium oxysporum* receptor protein 3-D structures were characterized, and their docking interaction analyses were carried out. The HMMER in silico tool identified novel anti-*Fusarium oxysporum* antimicrobial peptides with good performance in terms of accuracy, sensitivity, and specificity. These AMPs also displayed good physicochemical properties and bound with greater affinity to *Fusarium oxysporum* protein receptor PR-1-like Protein. The tendency of these AMPs to precisely detect *Fusarium oxysporum* PR-1-like Protein, Fpr1, would justify their use for the identification of the fungus. This study would enhance and facilitate the identification of *Fusarium oxysporum* to reduce problems associated with poor crop yield, economic losses, and decreased nutritional values of plants to keep up with the growing population.

## 1. Introduction

*Fusarium oxysporum* is a significant threat to agricultural production. Due to its considerable variation of morphological and physiological makeup resulting from an anamorphic species complex, it tends to escape detection [1]. This fungal pathogen is common globally in soils with the tendency to grow saprophytically or colonize plants. Its economic importance ranges from decreased crop yield, reduced nutritional and market value of farm produce to plants’ reduced resistance to the harsh environmental conditions [2]. The pathogen achieves this by blocking the plant’s water-conducting xylem tissues and subsequently producing germinating spores in the host [3]. The consequence of the aforementioned challenges is a negative effect on storage to an off-season period, causing scarcity for the ever-increasing population [4]. Hence, there is a need to prevent its menace for food abundance and security to meet the demand of our growing population.

The pathogenic strains of *Fusarium oxysporum* may cause infection such as severe vascular wilts and root rot diseases to not only *Phaseolus vulgaris* but also other plant hosts such as tomato, banana, cotton and legumes [5]. It is also being reported as an emerging human pathogen for immunocompromised patients [6]. Despite this tendency to infect different plant hosts, isolated *Fusarium oxysporum* strains only infect very few plant species during inoculation [6]. This inconsistency between field and laboratory conditions limits the fungal pathogen’s handling rate; hence, fast and dependable detection of this microbe is imperative with the end goal of appropriate and timely infection management measures.

Several mechanisms have been described that allow *Fusarium oxysporum* to recognize and penetrate a host and subdue the innate defenses and nutrient components of the host. The overall effect of the virulence factors produced determines both the infectious potential and severity of the disease caused [7]. Plants have the same innate immunity with the tendency to recognize pathogen-associated molecular patterns (PAMPs) or the presence of pathogenesis-related 1 (PR-1) proteins [8]. Of all the PR proteins, Fpr1 is the most highly expressed class with at least 10% of total Protein in infected hosts [7]. A secreted PR-1-like protein, Fpr1, in *Fusarium oxysporum* has been functionally characterized and its function is required for full virulence [9]. Despite the widespread use of PR-1-like Protein in *Fusarium oxysporum* as a virulence factor, its biochemical function and biological importance remain elusive.

Several methods are available to detect *Fusarium oxysporum* strains that are less accurate due to their similarity to other species. One detection method is based on the characteristic shape and size of macro-and microconidia with the presence and absence of chlamydospores [10]. There are also reports of observation of colony appearances, pigmentation, and growth rates on agar media, giving false positive results [11]. These methods’ reliability is questionable in terms of specificity as they can give false positive results with other *Fusarium* species [12]. Polymerase chain reaction with restriction fragment length polymorphism (PCR-RFLP) of the intergenic spacer (IGS) region is a gold standard and an emerging technique used for the identification of *Fusarium oxysporum* and has proven to be highly dependable for the differentiation of strains at the intra-specific level [13]. However, the difficulty of the continuous use of this method lies in the fact that *Fusarium oxysporum* has a cell wall that impedes its efficient lysis and liberation of DNA. However, this can lead to false negative PCR results, creating demand for more sensitive and accurate diagnostics [14].

Antimicrobial peptides (AMPs) are produced as part of the innate immune response, and they exhibit a broad spectrum of activities against pathogens [13]. They have several compensatory characteristics that make them unique, including cationic charge, hydrophobicity, and diverse structural forms [15]. They establish their activities by binding to membranes and cell walls, causing a non-enzymatic disruption with selectivity owing to the membrane composition of different microbes and cell types [16]. Application of AMPs has been reported recently for the early diagnosis of HIV using bioinformatics [17] with its molecular validation in a lateral flow device for Point-of-Care (POC) detection [18]. Thus, due to the AMP compensatory mechanism of actions highlighted above, the false negative results from existing methods such as PCR may be eliminated. Therefore, this present research work aims to use novel AMPs for the accurate detection of *Fusarium oxysporum* in *Phaseolus vulgaris*.

## 2. Materials and Methods

### 2.1. Data Collection

The anti-*Fusarium oxysporum* AMPs were retrieved from antimicrobial peptide databases such as Antimicrobial Peptides Database (APD3) [19,20] and Collection of Antimicrobial Peptides (CAMP) [21]. Thereafter, literature mining was carried out to confirm that all the recovered AMPs were either experimentally validated or predicted. Duplicate experimentally validated AMPs were then removed from the list using the Cluster Database at High Identity with Tolerance (CD-HIT) [22].

### 2.2. Division of Data into Training and Testing Datasets

The screened, experimentally validated AMPs were randomly partitioned into two subsets: 75% of the data were utilized as the training partition (to build each profile), while the remaining 25% was used for testing (including optimization/calibration of profiles).

### 2.3. Construction of AMPs Profiles

**Figure 1:**
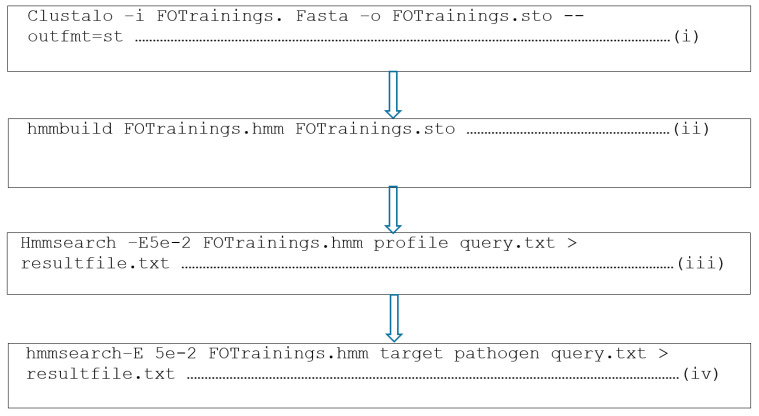
Flow chart of the HMMER command lines. The command line (i) in the flow chart above used “Clustalo” module of the HMMER software for the multiple alignment and GCG postscript output for the graphical printing of the AMPs. The command line hmmbuildin (ii) built the aligned sequences in (i) to enhance the construction of the profile by showing common motifs/signatures within the profile. The command line hmmsearchin (iii) evaluated the performance of the resulting constructed profile in (ii) by querying it on independent datasets. The command line (iv) allowed the identification of the anti-*Fusarium oxysporum* AMPs.

The Hidden Markov Models (HMMER) algorithm version 3.8 [23] was utilized to build specific pathogen-targeted profiles using the constructed datasets. The task was carried out on the terminal of the Ubuntu operating system version 12.04; (Canonical Ltd., London, UK) with the command line used for building the profile written in the flow chart (i) (Figure 1):

For the initial step, the training datasets of each target class were arranged by utilizing the Clustalo alignment tool [24].

### 2.4. Independent Profile Testing

The autonomous query of the profiles was performed in a step called “Query profiles”. The testing data were queried against each target profile utilizing the command line as stated above in the flow chart (iii) (Figure 1) with an E-value threshold of 0.05.

### 2.5. Performance Measurement of Each Profile

The statistical measures were carried out utilizing sensitivity, specificity, accuracy, and Matthews Correlation Coefficient as parameters. The parameters utilized are as described below where TP indicates true positive, TN indicates true negative, FP indicates false positive, and FN indicates false negative:

Percentage sensitivity of the anti-*Fusarium oxysporum* AMPs against a specific pathogen (testing sets) effectively predicted as anti-*Fusarium oxysporum* AMPs (positive). The equation of the sensitivity is written below as (1):

(1) $$\mathrm{Sensitivity}=\left(\frac{TP}{TP+FN}\right)\times 100$$

Percentage specificity of the non-anti-*Fusarium oxysporum* AMPs (negative sets) effectively predicted as non-anti-*Fusarium oxysporum* AMPs (negative). The equation of the specificity is written below as (2):

(2) $$\mathrm{Specificity}=\left(\frac{TN}{TN+FP}\right)\times 100$$

Percentage accuracy of the effectively predicted peptides (anti-*Fusarium oxysporum* AMPs and non-anti-*Fusarium oxysporum* AMPs). The equation of the accuracy is written below as (3):

(3) $$\mathrm{Accuracy}=\left(\frac{TP+TN}{TP+FP+TN+FN}\right)\times 100$$

Matthew’s correlation coefficient (MCC) measures the sensitivity and specificity. MCC = 0 is an indication of absolutely random prediction, while MCC = 1 means perfect prediction. See the Equation (4) as below:

(4) $$\mathrm{MCC}=\left(\frac{\left(TP\times TN)-(FN\times FP\right)}{\sqrt{\left(TP+FN)\times (TN+FP)\times (TP+FP)\times (TN+FN\right)}}\right)$$

### 2.6. Novel Putative Anti-Fusarium oxysporum AMPs Identification

Query of the proteome sequences were carried out by the respective profiles using the list of all proteome sequences collected from the Ensembl database (http://www.ensembl.org/index.html, accessed on 22 December 2019) [25] and the UniProt database (http://www.uniprot.org/, accessed on 23 December 2019) [26]. An E-value cut-off was set to 0.05 for the discovery of putative anti-*Fusarium oxysporum* AMPs. The accomplishment of this task was done using “hmmsearch” module of the HMMER software with the command line employed stated in the flow chart above (iv) (Figure 1).

Specific FOTrainings.hmm in the profile, target class query.txt representing the species scanned against the profile and resultfile.txt is the output file acquired after testing the species against the constructed *Fusarium oxysporum* (FO) profile.

### 2.7. Identification of Receptors

The gene for the receptor, PR-1-like protein, Fpr1, was identified for *Fusarium oxysporum* (isolate 4287 PR-1-like protein) and collected from the National Center for Biotechnology Information (NCBI) (https://www.ncbi.nlm.nih.gov/, accessed on 26 December 2019) [27], through literature mining. Thereafter, curation was performed to verify that the retrieved *Fusarium oxysporum* gene was complete. Thereafter, the translate tool of ExPAsy (https://web.expasy.org/translate/, accessed on 27 December 2019) [28] was used to translate the reading frame of the coding portion of the gene into protein. BLAST analysis was then performed using the UniProt interface (https://www.uniprot.org/help/uniprotkb, accessed on 23 January 2020) [26] for further assurance of specificity such that the PR-1-like protein of interest was specific for *Fusarium oxysporum*.

### 2.8. Evaluation of the Protein Receptor Model

The quality of the receptor PR-1-like protein, Fpr1, was analyzed using BIOVIA (https://www.3ds.com/products-services/biovia/, accessed on 27 January 2020) [29] online software that incorporates modeler tool (https://salilab.org/modeller, accessed on 27 January 2020) [30] by computing the validation/authentication of its residue regions during binding interaction.

### 2.9. Physicochemical Properties of the Putative Anti-Fusarium oxysporum AMPs and the Fusarium oxysporum Fpr1 Protein

Physicochemical properties of the anti-*Fusarium oxysporum* AMPs were calculated using the prediction interface of BACTIBASE (http://bactibase.pfba-lab-tun.org/physicochem, accessed on 31 January 2020) [31,32], DBAASP (https://dbaasp.org/, accessed on 31 January 2020) [33], and APD3 (https://wangapd3.com/main.php, accessed on 28 February 2020) [19,34] and the receptor PR-1-like protein, Fpr1, was carried out using ProtParam tool (http://web.expasy.org/protparam/, accessed on 28 March 2020) from the ExPAsy server [35] using the amino acid sequences of the PR-1-like protein, Fpr1, as input.

### 2.10. Structure Predictions of the Putative Anti-Fusarium oxysporum AMPs and Fusarium oxysporum Proteins

The I-TASSER (Iterative Threading ASSembly Refinement) server, which is an example of a de novo method of peptide or protein structure prediction, was used to generate the putative anti-*Fusarium oxysporum* AMPs as well as the *Fusarium oxysporum* PR-1-like protein, Fpr1, structures [36]. In brief, the prediction was performed by uploading each sequence onto the I-TASSER website [37]. PyMOL (Version 1.3), (Schrödinger, Inc., New York, NY, USA) was then used to visualize the 3-D structures of the AMPs and the protein receptor [38].

### 2.11. Interaction Analysis of the Putative Anti-Fusarium oxysporum AMPs and Fusarium oxysporum Protein

The PatchDock 1.3 web-server that enables the docking of the protein-small ligand molecule, available at http://bioinfo3d.cs.tau.ac.il/PatchDock/ (accessed on 31 March 2020) was used for the docking of the anti-*Fusarium oxysporum* AMPs to the *Fusarium oxysporum* PR-1-like protein, Fpr1 [39]. In brief, the PDB files generated from the I-TASSER for the 3-D structures of the anti-*Fusarium oxysporum* putative AMPs and the *Fusarium oxysporum* protein receptor were uploaded onto the PatchDock server. The complex formation with the interaction analysis between the anti-*Fusarium oxysporum* putative AMPs and the PR-1-like protein receptor was achieved using RasMol 2.7.5 Software (NextMove Software Ltd., Cambridge Science Park, UK) [40]. Subsequently, binding energy scores of the complex formed between the AMPs and the receptor protein were computed using HDock server (http://hdock.phys.hust.edu.cn/, accessed on 3 March 2021) [41].

## 3. Results

### 3.1. Data Collection

Experimentally validated anti-*Fusarium oxyporum* AMPs were collected from different databases—literature mining revealed that CAMP, APD3, DBAASP, and BACTIBASE had 2, 32, and 6 experimentally validated anti-*Fusarium oxysporum* antimicrobial peptides, respectively. After duplicate removal, a final list of 32 anti-*Fusarium oxysporum* AMPs was generated.

### 3.2. Profile Construction

The first step in the profile creation was the random partitioning of the experimentally validated AMPs (Table 1). HMMER was then used to cluster, build and search new AMPs with diagnostic relevance against *Fusarium oxysporum*.

### 3.3. Testing and Performance Measurement of the Profile

The profile was tested against a positive dataset which represented about a quarter of the dataset, from which the training dataset used for the construction of the profile was derived. In addition, the trained profile was scanned against a negative control dataset, made up of random fragments of 17236 neuropeptides, which had no recorded anti-*Fusarium oxysporum* activity (Table 2). The profile discriminated against the negative dataset, with only six of its eight positive datasets being a true positive. Thus, the purpose for dividing the AMPs into training and testing datasets was to ascertain the robustness and discriminatory power of the profile built by HMMER [17]. The performance result of the profile also showed that it was specific, accurate, and sensitive with a significant Matthews correlation coefficient (MCC).

### 3.4. Proteome Sequence Database Query and Discovery of Anti-Fusarium oxysporum AMPs

Scanning of the profile was carried out to identify novel anti-*Fusarium oxysporum* AMP sequences that adhered to the 0.05 E-value cut-off. This yielded 12 AMPs across all proteomes scanned that matched the profile (Table 3).

### 3.5. Receptor Identification

*Fusarium oxysporum* PR-1-like protein, Fpr1, was used as a receptor to serve as targets for the novel antimicrobial peptides for its detection in a plant host. The PR-1-like protein gene (Fpr1) was identified for *Fusarium oxysporum* from the National Centre for Bioinformatics Institute (NCBI) database. It was translated using the ExPAsy translate tool using the coding unit of the gene. It was projected that this PR-1-like protein is potentially relevant in detecting *Fusarium oxysporum* because of its compensatory advantages ranging from production in very high concentration to accessibility for detection [42].

### 3.6. 3-D Model Structure Validation

**Figure 2:**
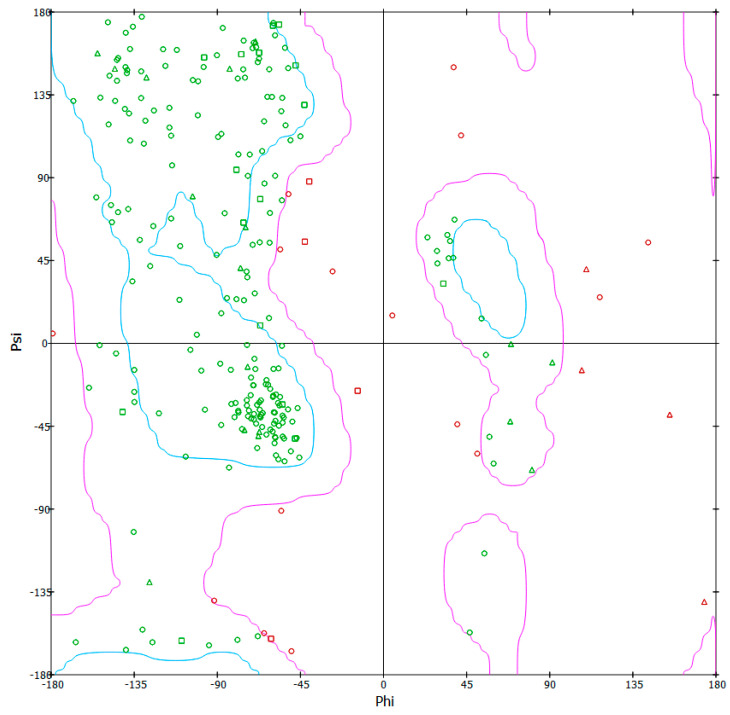
BIOVIA result for modeled PR-1-like Protein (Fpr1) of *Fusarium oxysporum* using the generated model from I-TASSER. Residues in most favored regions (upper first box on the left), residues in additional allowed regions (lower box on the left), and residues in generously allowed regions (upper box on the right).

BIOVIA, an online tool for verification of structure using modeler Ramachandran plot [29], was used to validate and evaluate the protein model’s quality (Figure 2). The model structure of the PR-1-like protein has 91.8% residues in the most favored region, 7.6% residues in an additional allowed region, 0.2% residues in a generously allowed region, and 0.4% residues in the disallowed regions.

### 3.7. Physicochemical Analysis of the Anti-Fusarium oxysporum AMPs and Fusarium oxysporum PR-1-Like Protein

Physicochemical features such as molecular weight amino acid composition, hydrophobicity, Boman index, net charge, isoelectric potential, and half-life were used to evaluate the anti-*Fusarium oxysporum* AMPs. From Table 4, BOMK-1 to 9, including 11 and 12, had glycine as a common amino acid. BOMK-7 and 12 had, in addition to glycine, alanine, and cysteine, respectively. BOMK-10 and 13 had cysteine. All the AMPs had significant hydrophobicity values between 32 and 42, with the lowest value observed for BOMK-9. The hydrophobicity result was the percentage of the total hydrophobic amino acids in the peptides as calculated from APD3 and BACTIBASE. All the AMPs had positive charges with the exception of BOMK-11, 12, and 13, which had negative and neutral charges, respectively. The isoelectric point for the AMPs was between 3.75 and 8.70, while the Boman Index was observed to be between 0.26 and 2.04. Lastly, the AMPs had significant half-lives with the lowest value observed for BOMK-10 (1.3 h).

In Table 5, PR-1-like Protein, Fpr1, had common amino acid alanine, a molecular weight of 95,472.44 Da, significant hydrophobicity, an isoelectric point of 10.00, and a half-life of 1.1 h in mammals with aliphatic and instability indices of 82.72 and 71.04 respectively.

### 3.8. Structure Prediction and Docking

The structure of the anti-*Fusarium oxysporum* AMPs was predicted using certain parameters such as C score, TM score, and RSMD as indicators (Table 6). All AMPs had significant C score values, TM scores, and RSMD, where the C score between −5 and 2 indicates structural prediction with high confidence. All the protein and AMP structures were predicted with high confidence because they had existing templates for their database validation. The TM scores for the AMPs and the receptor protein were >0.5, indicating correct topology for the anti-*Fusarium oxysporum* AMPs and *Fusarium oxysporum* protein, while the RSMD for these AMPs and receptor protein were between 2 and 4 Å indicating good prediction except for BOMK-4 and BOMK-11, indicating ideal predictions.

**Figure 3:**
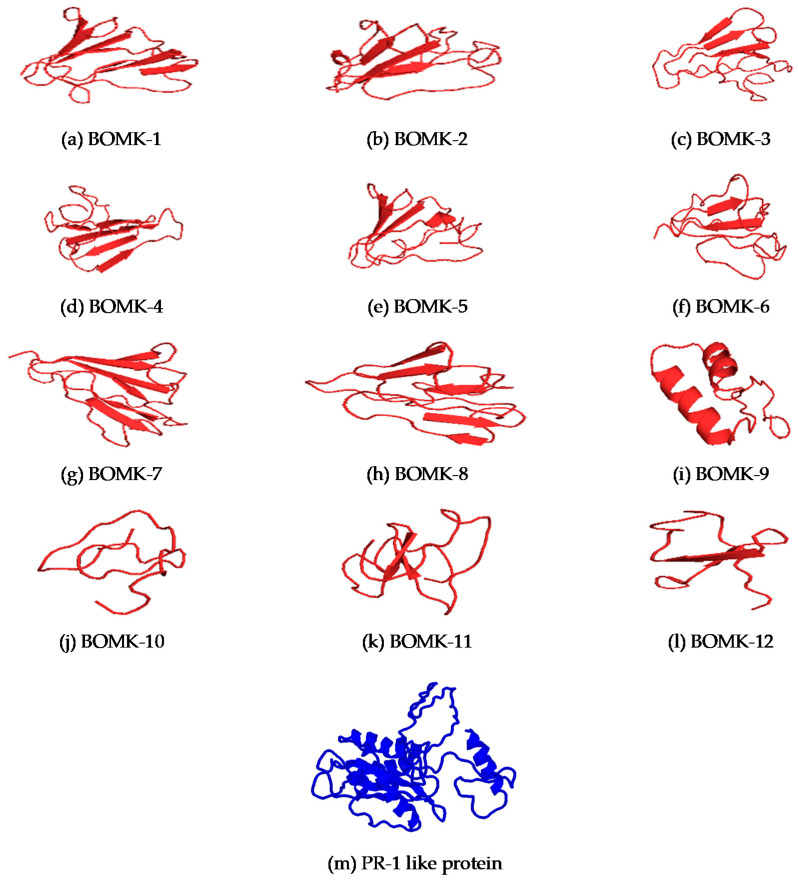
3D structures of the anti-Fusarium oxysporum AMPs and Fusarium oxysporum PR-1-like protein, Fpr1, as determined by I-TASSER. 3D structures of anti-Fusarium oxysporum AMPs (**a**–**l**) represented in red and Fusarium oxysporum Fpr1 (**m**) represented in blue.

Representative output images from the I-TASSER server after predicting the 3-D structures of the anti-*Fusarium oxysporum* AMPs (ligands) and the protein receptors are indicated in Figure 3.

### 3.9. Protein-Peptide Interaction between Anti-Fusarium oxysporum and Fusarium oxysporum Fpr1

The docking results of the complex between the putative AMPs and PR-1-like protein, Fpr1, is displayed in Table 7. All the AMPs showed good binding affinity to PR-1-like protein, Fpr1, greater than 8741 [43]. It was observed that BOMK-10, 12, 6, and 8 had the highest binding scores with the lowest observed for BOMK-11 and 9, respectively, using PatchDock. These binding geometry scores are indicators of high detection of the *Fusarium oxysporum* Frp1. Using binding energy scores from the HDock server, it was observed that all the anti-*Fusarium oxysporum* displayed high binding energy with BOMK-7 having the highest binding energy, followed by BOMK-3 and -5. The complex formation occurred at the *Fusarium oxysporum* PR-1-like protein, Fpr1, the most favored regions.

**Figure 4:**
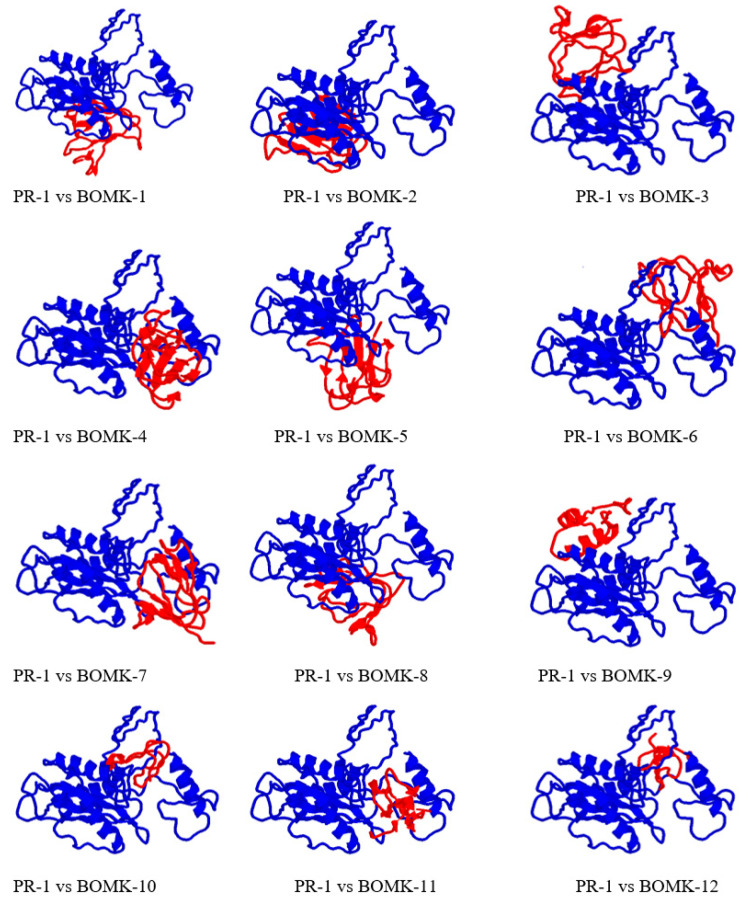
Docking interaction analysis of the anti-*Fusarium oxysporum* AMPs with *Fusarium oxysporum* PR-1 like protein, Fpr1. The blue color represents PR-1 like protein (Fpr1), the red is the AMPs.

The structural complex of the docking results between the PR-1-like protein, Fpr1, of *Fusarium oxysporum,* and anti-*Fusarium oxysporum* AMPs downloaded as PDB files and visualized using RasMol software is shown in Figure 4 in which the blue represents Fpr1 and the anti-*Fusarium oxysporum* AMPs are shown in red. BOMK-1 and -2 bound at the same position, BOMK-3 and -9 bound at the same position, BOMK-4 and -7 bound at the same position, BOMK-5 and -8 bound at the same position, while BOMK-6 bound at the different orientation of the PR-1-like protein from others.

## 4. Discussion

### 4.1. Data Retrieval and Profile Construction of the Anti-Fusarium oxysporum AMPs

Anti-*Fusarium oxysporum* AMPs that have been experimentally approved were recovered from different databases since they have been demonstrated to possess activities against *Fusarium oxysporum* by utilizing the agar dilution or broth microdilution techniques with the minimum inhibitory concentration (MIC) assay [44]. The rundown of anti-*Fusarium oxysporum* AMPs from the databases was recovered after eliminating duplicates to take into account specific species/pathogen profile creation.

The training dataset was made up of ¾ of the retrieved peptides required to prepare the algorithm to test whether the functionally critical amino acid consensus is preserved. After this, multiple alignments were created utilizing HMMER, which keeps the profile from being sensitive to little misalignments and report significant E-values. This allows the tendency to capture sequence diversity since the AMPs were obtained from various life forms [45]. Clusters by HMMER likewise permit a minimum measure of closeness between all peptides.

### 4.2. Testing of the Profiles

The profile constructed utilizing the training dataset was applied against the held out, positive testing dataset to assess the trained model’s ability to recognize and distinguish this subset of AMPs. Since experimentally confirmed AMPs were utilized, the assumption will be that the profiles developed should have the ability to identify different sequences with similar action and reject those that have no anti-*Fusarium oxysporum* activity. The utilization of a negative dataset (neuropeptides) was done to affirm whether the prepared profiles would discriminate non-anti-*Fusarium oxysporum* peptides. The utilization of random sequences as a negative dataset is a regularly utilized method [46].

The assessment of the autonomous profile testing was accomplished utilizing the TP, FP, TN, and FN measures as inputs to the sensitivity, specificity, accuracy, and MCC descriptive statistics. The HMMER E-value cut-off was set to 0.05 to improve the discovery capacity of the profile between the true positive anti-*Fusarium oxysporum* AMP and false negative anti-*Fusarium oxysporum* AMPs. The FO (against *Fusarium oxysporum*) profile had six of its eight positive datasets as true positive, bringing about high sensitivity. The MCC is considered to give the best performance estimation of profiles since it provides the best connection between sensitivity, specificity, and accuracy [47]. The high specificity implies the profile had no comparable capacity with different profiles. The accuracy result was exceptionally high for all the profiles demonstrating the elimination of errors by invalidating misclassified AMPs from both positive and negative datasets. The MCC value “0.5 to 1” relates to correct prediction, while “0” focuses on an irregular forecast. MCC is considered the most robust estimation for assessing the prediction of profile performance. Along these lines, the FO profile shows the right prediction.

HMMER utilized a default E-value of 0.05 for each hit viewed as a true positive. The anti-*Fusarium oxysporum* profile yielded true positives with E-values lower than 0.05 showing that there was just a 5% possibility that the hit was false or arbitrary. This outcome concurs with the work of Bhadra, Yan [48] where performance was analyzed in terms of accuracy, specificity, sensitivity, and Matthews Correlation Coefficient (MCC) utilizing benchmark datasets as information sources.

### 4.3. Proteome Sequence Database Query and Discovery of Anti-Fusarium oxysporum AMPs

The discovery stage was to identify novel AMPs with the ability to detect anti-*Fusarium oxysporum* in infected plant tissues. This was carried out to discover the AMPs with the same signature/motif as the input sequences. A final list of twelve AMPs was identified, and the AMPs were categorized according to their E-values, with those having the smallest E-values considered the most probable putative anti-*Fusarium oxysporum* AMPs. There was a very small likelihood that these peptides were incorrectly predicted to be anti-*Fusarium oxysporum* AMPs.

### 4.4. Receptor Identification

PR-1-like protein, Fpr1, is a protein of *Fusarium oxysporum* used for proteolytic processing and activation of secreted effectors by fungal and plant host proteases (Avr4). It is a well-characterized type of PR-1 like protein in humans that has been associated with rudimentary biological processes such as cancer, reproduction, and immune response, which are inferred indirectly based on gene expression, localization in specific cell types (glioma or sperm cells), or in response to certain stimuli (pathogen attack) rather than by firm genetic evidence [49]. The highly specific role of PR-1-like protein, Fpr1, during fungus-host interaction makes it a promising target for *Fusarium oxysporum* detection.

From Table 4, PR-1-like protein, Fpr1, of *Fusarium oxysporum* is a moderately stable protein. The 3-D model structure validation using BIOVIA also supports its use because of its high quality in terms of the distribution of amino acid residues (Table 1), and thus, this justifies its use for the detection of this fungus [50,51].

### 4.5. Physicochemical Analysis

The physicochemical properties of the putative AMPs were resolved by utilizing APD and BACTIBASE to guarantee that the distinguished sequences adjust to other AMPs dependent on the qualities estimated. The hydrophobicity result, which was lower than 30% is not an ideal physicochemical parameter [52]. Peptides with higher hydrophobicity would penetrate further into the cell’s hydrophobic center to exert their antimicrobial effects through several mechanisms exhibited by the peptides [53]. All the anti-*Fusarium oxysporum* AMPs which were positively charged demonstrated congruity of ideal AMPs with improved antimicrobial activity. Notwithstanding, the absence of the positive charge in the net charge of BOMK-10, 11, and 12 does not imply a lack of antimicrobial activity since some negatively charged AMPs have quite recently been accounted for. For example, the surfactant-related anionic peptide in the APD3 database (AP00528) with a net charge of −5 has an anti-bacterial action, and maximin H5 with a charge between −1 and −7 has a bacterial growth restraint action against *Listeria* *monocytogenes* [54]. The range of isoelectric values of the AMPs between 3.75 and 8.70 shows characteristic solubility properties for the AMPs in acid and alkaline media despite the variability of charges [55]. The isoelectric point (pI) of peptides is a component of individual amino acids in both original structures. A negative Boman index is said to be related to a more hydrophobic peptide, demonstrating a high protein binding potential, while a more hydrophilic peptide will, in general, have a more positive index [56]. In any case, the propensity of certain peptides to be positive in their Boman index values has been associated with the capacity to identify HIV in a lateral flow device [17].

The physicochemical parameters of the PR-1-like protein, Fpr1 (Table 5) indicate that it is an ideal candidate for the identification of *Fusarium oxysporum* in terms of stability (as indicated by the instability index), with alanine, valine, isoleucine, and leucine being the most abundant contributors to the aliphatic side chains resulting in an increased thermo-stability (with alanine being the most abundant).

### 4.6. Structure Prediction and Docking Interaction Analysis of the Putative Anti-Fusarium oxysporum and Fusarium oxysporum PR-1-Like Protein

The structure prediction of the AMPs and the *Fusarium oxysporum* protein receptor was analyzed in Table 6. The C-score is a certainty score for assessing the nature of anticipated models by I-TASSER. Its assessment depends on the significance of threading template arrangements and the combination parameters of the structure assembly simulations, which are regularly in the scope of −5 to 2. A C-score inside this scope of values connotes a model with high certainty [57]. The prediction of the models of the anti-*Fusarium oxysporum* AMPs and the *Fusarium oxysporum* Fpr1 had high confidence in terms of the templates used for their prediction.

On the other hand, TM-score is a proposed scale for estimating the basic convergence/similarity between two structures [58]. A TM-score of >0.5 indicates a correct topology model, and a TM-score of <0.17 means a random similarity. All the AMPs, including the receptor protein, had the correct topology without arbitrary similarity to any other models.

Even though there is certifiably not a characterized RMSD value for 3-D structure prediction, an RMSD estimation of 2–4 Å is viewed as acceptable, and an RMSD of ≤1 Å is considered to be ideal. All models had ideal qualities for RMSD. The results similarly showed that all the AMPs different secondary structures, including α-helices, parallel β-sheet, anti-parallel β-sheet, extended, and loop conformational structures. The outcomes observed associate with the various structural conformations displayed by known AMPs. Examples of known AMPs and their structures include tachyplesin from horseshoe crabs and bovine lactoferricin, which have beta-sheet structures [59]; magainin analog and melittin having alpha-helical conformations [60]. Consequently, the peptides can be considered bona fide AMPs. In any case, the AMPs identified in this study are thought to be putative anti-*Fusarium oxysporum* peptides because of the absence of experimental proof for these molecules at present.

Utilizing the binding geometry scores in Table 7, all the putative AMPs indicated a huge binding affinity to the PR-1-like protein of *Fusarium oxysporum*. The AMPs also displayed high binding energy scores with PR-1-like protein, Fpr1, with the AMPs having the most noteworthy inclination to identify the fungus. PatchDock and HDock servers use the scoring function as provided in this research to sample ligands’ conformations on the protein receptor [39,41]. The HDock server, for instance, uses a flexible receptor molecular docking approach to estimate and assess the non-bonded (electrostatic and van der Waals) interactions utilizing the classical force-field-based scoring function [41]. The utilization of HMMER for the discovery of putative AMPs in this research can be used to identify *Fusarium oxysporum* in plants by utilizing PR-1-like protein, Fpr1, as a target under high sensitivity, specificity, and accuracy.

## 5. Conclusions

This research identified novel AMPs for the potential diagnosis of *Fusarium oxysporum* using HMMER in silico technology, where 12 anti-*Fusarium oxysporum* AMPs were generated. The putative anti-*Fusarium oxysporum* AMPs showed conformity to other known AMPs in terms of their physicochemical characteristics. This diagnostic system’s primary goal is to ease the search and identify a standard reference for a biomarker for early detection of the fungus to solve the current problem, which leads to the reduction of crop yield, market value, and nutritional value of crop plants, including *Phaseolus vulgaris*. AMPs have demonstrated incredible promise in evading the downsides related to the current diagnostic systems of this fungus. This research work could be pursued for molecular validation through the binding of these AMPs with the PR-1-like protein, Fpr1, using an “on/off” binding experiment in an LFD setting to develop a prototype with these specific AMPs conjugated to gold nanoparticles (AuNPs) to accurately and sensitively detect the fungal pathogen within plant samples.

## Figures and Tables

**Table 1:** Profile creation by HMMER.

Profiles	Training Datasets	Testing Datasets	Total
FO	24	8	32

**Table 2:** Independent testing of the profile.

**True Positive**	**False Negative**	**True Negative**	**False Positive**
**6**	2	17,236	0
**Sensitivity (%)**	**Specificity (%)**	**Accuracy (%)**	**MCC**
**75**	100	99.99	0.87

**Table 3:** Discovery of anti-*Fusarium oxysporum* AMPs.

Organism	Name	AMPs	Number of Amino Acid Residues	Bit Scores	E Values
***Selaginella moellendorffii***	BOMK-1	AlaTrpAlaGlyProGlyCysAsnAsnArgLeu----------ValGlyAlaSerGlnHisGlyGlyTyrSerPheAlaTyrGlnGlyGlnThrAlaAlaAlaTyrAsnThrAlaAsnCysArgGlyValAlaHisThrArgPheSerSerLysGlyGluCysLysSerGlySerValGlnAspCysSerGlyPheGlyTrpArgSerIlePheIleGlnCys	80	35.3	5 × 10^−8^
***Selaginella moellendorffii***	BOMK-2	TrpAlaGlyProGlyCysAsnAsnArgLeuGlu----------GlyAlaSerGlnHisGlyGlyTyrSerPheAlaTyrGlnGlyGlnThrAlaAlaAlaTyrAsnThrAlaAsnCysGlnGlyValAlaHisThrArgPheSerArgLysGlyGluCysLysSerGlySerValGlnAspCysSerGlyPheGlyTrpAsnSerPhePheIleGlnCys	80	32.8	3.3 × 10^−7^
***Selaginella moellendorffii***	BOMK-3	ThrTrpAlaGlyProGlyCysAsnAsnArgLeu----------ValGlyAlaSerGlnHisGlyGlyTyrSerPheGlyTyrGlnGlyGlnThrAlaAlaAlaTyrAsnThrAlaAsnCysGlnGlyValAlaHisThrArgPheSerArgLysGlyGluCysLysSerGlySerValGlnAspCysSerGlyPheGlyTrpAsnSerPhePheIleGlnCys	80	32.7	3.6 × 10^−7^
***Selaginella moellendorffii***	BOMK-4	AlaTrpAlaGlyProGlyCysAsnAsnValLeu----------ValArgAlaSerGlnHisGlyGlyTyrSerPheValTyrGlnGlyGlnThrAlaAlaAlaTyrAsnThrAlaAsnCysArgGlyValAlaHisThrArgPheSerArgLysGlyGluCysLysSerGlySerValGlnAspCysSerGlyPheGlyTrpAsnSerPhePheIleGlnCys	80	31.1	1.1 × 10^−6^
***Selaginella moellendorffii***	BOMK-5	ThrTrpAlaGlyProGlyCysAsnAsnGlnArg----------ValGlyAlaSerGlnHisGlyGlyTyrSerPheGlyTyrGlnGlyGlnThrAlaAlaAlaTyrAsnThrAlaAsnCysGlnGlyValAlaGlnThrArgPheSerAlaLysGlyGluCysLysSerGlySerValGlnAspCysSerGlyPheGlyTrpAsnSerPhePheIleGlnCys	80	27.6	1.4 × 10^−5^
***Selaginella moellendorffii***	BOMK-6	TrpAlaGlyProGlyCysAsnAsnTrpLeuGlu----------AlaSerGlnHisGlyGlyTyrSerValAlaTyrLeuGlyHisAlaAlaAlaAlaTyrAsnThrAlaAsnCysGlnGlyValAlaGlnArgTrpPheArgArgLysGlyHisCysSerSerGlyCysAlaSerGluCysGluGlyPheArgTrpAsnSerIlePheIleGlnCysSerSer	80	26.4	3.6 × 10^−5^
***Selaginella moellendorffii***	BOMK-7	TrpAlaGlyProGlyGlyAsnAsnArgLeuGlu----------AlaSerGlnHisGlyGlyTyrSerValValTyrLeuGlyHisAlaAlaAlaAlaTyrAsnThrAlaAsnCysGlnGlyValAlaGlnArgTrpPheArgArgLysGlyHisCysSerSerGlyCysAlaSerGluCysGluGlyPheArgTrpAsnSerIlePheIleGlnCysSerSer	80	25.0	0.00011
***Setaria italic***	BOMK-8	ThrSerTrpAlaGlyProGlyCysSerGlyGln----------AsnLeuGlnPheTyrAspGlyGlnGluLysSerTyrGlnGlyGlnThrAlaArgLeuTyrThrGluThrGlyCysAlaGlyThrSerTyrLeuValPheGluAspThrGlnAlaCysGlySerGlyCysAlaSerGluCysGluAspPheGlyTrpArgSerIle	75	21.8	0.00073
***Oryza sativum***	BOMK-9	LysIleGlnValGluAlaLysSerCysCysProGly----------TyrAsnSerCysArgPheAlaGlyGlySerArgAspThrCysAlaLysLeuSerGlyCysLysIleValCysAspGlyAsnCysLysProProTyr	54	23.5	0.00079
***Zea mays***	BOMK-10	GlyGlyHisProAspGlyAlaIleProCysGlyGlu----------ValPheGlyCysArgGlyTrpGlyTyrCysGlu	33	19.8	0.0037
***Solanum lycopersicum***	BOMK-11	AlaGlnGlnCysGlyIleGlnAlaGlyGlyAla----------PheGlyTyrCysGlyThrThrAlaThrAlaTyrCysGlyProGlyCysGlnSerGlnCys	41	16.0	0.026
***Arabidopsis thaliana***	BOMK-12	ValGlnGluTyrGlyCysProAsnCysLysArg----------GlyGluLeuValMetGluCysAsnLys	30	17.7	0.034

BOMK1–12: Anti-*Fusarium oxysporum* AMPs, “-” means specific amino acid residues which will be made available on request.

**Table 4:** Physicochemical properties of the anti-*Fusarium oxysporum* AMPs.

AMP	Mol. Mass (Da)	Common Amino Acids	Hydrophobicity (%)	Isoelectric Point	Boman Index (Kcal/mol)	Charge	Half-Life (h)
BOMK-1	8651.30	G	35	8.70	1.89	+5	4.4
BOMK-2	8531.35	G	35	8.28	1.69	+3	2.8
BOMK-3	8618.44	G	33	8.28	1.71	+3	7.2
BOMK-4	8700.36	G	37	8.49	1.70	+4	4.4
BOMK-5	8509.84	G	34	8.28	1.51	+3	7.2
BOMK-6	8900.35	GA	40	8.40	1.63	+4	2.8
BOMK-7	8852.32	G	37	8.70	1.81	+5	2.8
BOMK-8	8121.76	G	32	3.88	1.42	−5	7.2
BOMK-9	5796.17	C	35	8.70	1.72	+5	1.3
BOMK-10	3376.98	G	42	4.42	0.26	−2	30
BOMK-11	4042.34	GC	41	3.75	0.45	−1	4.4
BOMK-12	3537.55	C	40	7.08	2.04	0	100

BOMK1–12: Anti-*Fusarium oxysporum* AMPs.

**Table 5:** Physicochemical of the PR-1-like protein, Fpr1, of *Fusarium oxysporum*.

Receptor	M. wt (Da)	Common Amino Acid	Hydrophobicity (%)	Isoelectric Point	Instability Index	Aliphatic Index	Half-Life (Hours)
PR-1-like protein	95,472.44	SLP	40	10.00	71.04	82.72	1.1

**Table 6:** Structure prediction of the anti-*Fusarium oxysporum* AMPs and PR-1-like protein, Fpr1, from I-TASSER.

AMPs	C Score	TM Score	RSMD (Å)
BOMK-1	0.84	0.83 ± 0.08	2.0 ± 1.6
BOMK-2	0.49	0.78 ± 0.10	2.6 ± 1.9
BOMK-3	0.83	0.83 ± 0.08	2.0 ± 1.6
BOMK-4	0.88	0.83 ± 0.08	1.9 ± 1.6
BOMK-5	0.74	0.81 ± 0.09	2.2 ± 1.7
BOMK-6	0.77	0.82 ± 0.09	2.1 ± 1.7
BOMK-7	0.73	0.81 ± 0.09	2.3 ± 1.8
BOMK-8	0.67	0.80 ± 0.09	2.2 ± 1.7
BOMK-9	0.42	0.66 ± 0.13	3.6 ± 2.5
BOMK-10	0.86	0.61 ± 0.14	3.5 ± 2.4
BOMK-11	0.58	0.79 ± 0.09	1.4 ± 1.3
BOMK-12	−0.75	0.62 ± 0.14	3.3 ± 2.3
PR-1-like protein	−1.44	0.54 ± 0.15	9.1 ± 4.6

**Table 7:** PATCHDOCK results for each AMPs and PR-1-like protein with the geometry binding affinity.

AMPs	Pathdock Geometry Binding Affinity Scores	HDock Binding Energy Scores (Kcal/mol)
BOMK−1	12,828	−254.09
BOMK−2	12,976	−238.30
BOMK−3	13,776	−263.43
BOMK−4	12,652	−244.88
BOMK−5	13,688	−263.63
BOMK−6	14,334	−242.95
BOMK−7	12,776	−273.34
BOMK−8	14,016	−213.09
BOMK−9	11,468	−239.40
BOMK−10	14,806	−230.06
BOMK−11	9958	−220.11
BOMK−12	14,420	−236.68

## Data Availability

All data generated or analyzed during this study are included in this published article.
